# Age-Related Macular Degeneration: Cellular and Molecular Signaling Mechanisms

**DOI:** 10.3390/ijms26136174

**Published:** 2025-06-26

**Authors:** Feipeng Jiang, Jier Ma, Chunyan Lei, Yun Zhang, Meixia Zhang

**Affiliations:** Department of Ophthalmology and Research Laboratory of Macular Disease, West China Hospital, Sichuan University, Chengdu 610041, China; feipengj@163.com (F.J.); 13868112611@163.com (J.M.);

**Keywords:** age-related macular degeneration, oxidative stress, inflammation, immune dysregulation, aberrant lipid metabolism, angiogenesis, molecular mechanisms

## Abstract

Age-related macular degeneration (AMD) is a progressive retinal disorder and a leading cause of irreversible blindness among elderly individuals, impacting millions of people globally. This review synthesizes the current understanding of the cellular and molecular signaling mechanisms driving AMD, with a focus on the distinct pathophysiological features of dry and wet AMD subtypes. Key mechanisms include oxidative stress, inflammation, lipid metabolism dysregulation, and immune dysregulation, all of which converge on the retinal pigment epithelium (RPE) as a central player in disease initiation and progression. In dry AMD, oxidative damage, mitochondrial dysfunction, and lipofuscin accumulation impair RPE function, contributing to drusen formation and geographic atrophy. In wet AMD, vascular endothelial growth factor-mediated angiogenesis, coupled with inflammation and endothelial metabolic reprogramming, drives choroidal neovascularization. This article integrates findings from multiomics approaches and highlights the potential of artificial intelligence in elucidating AMD pathogenesis and advancing personalized therapies. Future research directions emphasize targeting these molecular pathways to develop innovative treatments, offering hope for improved management of this debilitating condition.

## 1. Introduction

Age-related macular degeneration (AMD) is a leading cause of vision loss in individuals over 55 years of age in developed countries [1]. With an aging global population, the prevalence of AMD is projected to rise, with estimates suggesting that by 2040, approximately 288 million people will be affected worldwide [1], posing significant challenges to healthcare systems and quality of life [2]. The disease manifests in two primary forms: dry AMD, characterized by gradual atrophy of the retinal pigment epithelium (RPE) and photoreceptors, and wet AMD, characterized by aberrant choroidal neovascularization (CNV) [3]. These pathological changes disrupt the retina’s neurovascular–immune microenvironment, resulting in progressive visual impairment.

The pathophysiology of AMD is complex and is driven by oxidative stress, chronic inflammation, lipid metabolism abnormalities, and immune dysregulation [4]. The RPE, a critical layer supporting photoreceptor function and retinal homeostasis, is particularly susceptible. In dry AMD, oxidative damage and lipid-rich drusen impair RPE integrity [4], whereas in wet AMD, inflammatory and angiogenic processes accelerate disease progression [5]. Although treatments such as anti-vascular endothelial growth factor (VEGF) agents for wet AMD and emerging complement inhibitors for dry AMD have advanced care, limitations persist, including inconsistent treatment responses and the absence of effective early-stage interventions.

This review integrates current findings on the cellular and molecular mechanisms of AMD, focusing on the interplay among oxidative stress, inflammation, lipid dysregulation, and angiogenesis. Drawing on insights from multiomics and advanced imaging, this review examines the roles of the RPE, immune system, and metabolic pathways in disease pathogenesis. The review is structured to first explore the RPE and oxidative stress, followed by investigations of inflammation, lipid metabolism, and the angiogenic mechanisms specific to wet AMD. Finally, we outline future directions, emphasizing the potential of multiomics and artificial intelligence to enhance AMD diagnosis and treatment. Our goal is to provide a clear framework for understanding AMD and inspire innovative treatment approaches.

## 2. Oxidative Stress

### 2.1. Sources of Oxidative Stress

**Figure 1:**
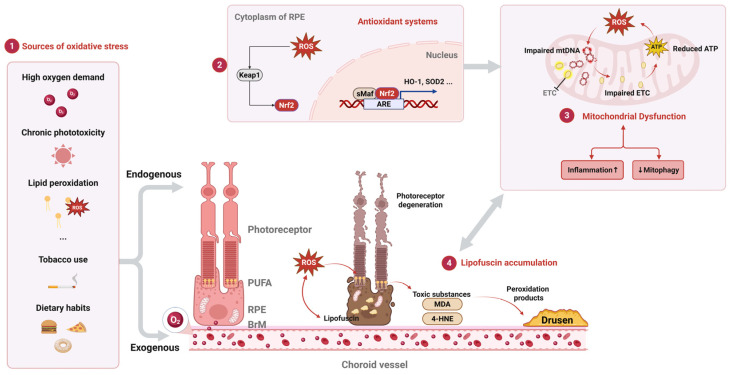
Oxidative stress pathways and RPE dysfunction in AMD. Schematic depicting key sources of oxidative stress (**part 1**), antioxidant defense mechanisms (including Keap1/Nrf2/ARE pathway) (**part 2**), and consequences of oxidative damage in the retinal pigment epithelium (RPE) during AMD pathogenesis (**part 3**). Mitochondrial dysfunction (**part 4**): lipofuscin accumulation. ROS, reactive oxygen species; PUFA, polyunsaturated fatty acids; RPE, retinal pigment epithelium; BrM, Bruch’s membrane; Nrf2, nuclear factor erythroid 2-related factor 2; Keap1, Kelch-like ECH-associated protein 1; ARE, antioxidant response element; sMaf, small Maf protein; HO-1, heme oxygenase-1; SOD2, superoxide dismutase 2; mtDNA, mitochondrial DNA; ETC, electron transport chain; ATP, adenosine triphosphate; 4-HNE, 4-hydroxynonenal; MDA, malondialdehyde. Upward arrow (↑) indicates enhancement/increase; downward arrow (↓) indicates reduction/decrease.

The retina experiences significant oxidative stress from both endogenous and exogenous sources (Figure 1). Endogenous stressors arise from the unique anatomical and metabolic properties of the retina, which create a microenvironment highly conducive to reactive oxygen species (ROS) generation. Endogenous stressors: (1) High oxygen demand: The outer retina–RPE complex sustains elevated metabolic activity, particularly in photoreceptors, which demand high oxygen levels and consequently produce substantial amounts of ROS [6]. (2) Chronic phototoxicity: Prolonged exposure to ultraviolet/blue light combined with abundant photosensitizers (e.g., retinaldehyde, zeaxanthin) in the neurosensory retina and RPE drives photochemical oxidation. (3) Lipid peroxidation-prone membranes: Photoreceptor outer segments contain high concentrations of polyunsaturated fatty acids (PUFAs). These lipids act as primary targets for ROS-mediated peroxidation, initiating cytotoxic chain reactions that directly impair RPE function [6].

Exogenous factors further exacerbate the retina’s oxidative load. Cigarette smoke (Figure 1), a major contributor [7], delivers more than 4700 chemicals—including approximately 1015 free radicals per puff—directly into systemic circulation [8]. These oxidants overwhelm endogenous antioxidant defenses, depleting critical molecules such as ascorbic acid and glutathione while also disrupting protein thiols and inducing oxidative modifications to DNA, lipids, and proteins. Although a direct causal link between smoking and AMD remains unconfirmed, its role in amplifying systemic oxidative stress is well documented [9]. Dietary habits also influence retinal oxidative balance: high-fat diets, particularly those rich in saturated fats, promote lipoprotein oxidation and foster a pro-oxidative microenvironment. While less impactful than smoking, such dietary patterns represent a modifiable risk factor for AMD, underscoring the interplay between lifestyle choices and retinal health [9].

### 2.2. Antioxidant Systems

The retina employs a sophisticated network of antioxidant defenses to counteract its inherent oxidative burden. These systems spatially and temporally regulate ROS to prevent widespread damage. For example, oxidant-producing enzymes localize near their molecular targets, whereas oxidants such as hydrogen peroxide (H_2_O_2_) enter cells through regulated pathways such as aquaporin channels, ensuring controlled activity [10]. The transient nature of ROS—evident in their short half-life and brief production bursts, such as during NADPH oxidase (NOX)-dependent signaling—further confines their effects to specific cellular compartments. This spatial restriction allows the localized neutralization of excess ROS before they inflict systemic harm.

At the cellular level [11], oxidative stress triggers a robust antioxidant response in RPE cells [12], mediated by the Keap1/Nrf2 pathway (Figure 1, part 2) [13]. The transcription factor nuclear factor erythroid 2-related factor 2 (Nrf2) serves as a master regulator of cellular antioxidant defenses. Under basal conditions, Nrf2 is constitutively bound to Kelch-like ECH-associated protein 1 (KEAP1), which targets it for ubiquitin-mediated degradation. However, elevated oxidative stress disrupts this interaction, liberating Nrf2. Subsequently, Nrf2 translocates to the nucleus where it heterodimerizes with transcriptional coactivators, such as small Maf proteins (sMAF). This complex binds to antioxidant response elements (AREs) within the promoter regions of target genes, initiating their transcription. Key antioxidant genes activated by Nrf2 include heme oxygenase-1 (HO-1), which degrades heme to release iron and the antioxidant biliverdin, and superoxide dismutase 2 (SOD2), which catalyzes the conversion of superoxide anion into hydrogen peroxide (H_2_O_2_). In AMD, this critical defense mechanism is compromised by the dysregulation of Nrf2 signaling. This dysregulation, often resulting from enhanced KEAP1-mediated ubiquitination or the impaired nuclear translocation of Nrf2, renders RPE cells vulnerable to an unchecked accumulation of ROS [14]. Experimental evidence underscores the critical role of Nrf2: Nrf2-deficient mice exhibit accelerated retinal degeneration, whereas the pharmacological activation of Nrf2—through the use of compounds such as sulforaphane—reduces oxidative injury and preserves retinal integrity, suggesting therapeutic potential for AMD [14].

Notably, the Keap1/Nrf2 pathway intersects with autophagy, a cellular recycling process that degrades oxidatively damaged components. Autophagy serves as a secondary protective mechanism [15], clearing dysfunctional organelles and protein aggregates generated by oxidative stress [16]. Compromised autophagy in RPE cells disrupts this quality control system [17], leading to the accumulation of toxic debris and contributing to AMD-like pathology in preclinical models [18].

### 2.3. Oxidative Damage in the RPE

RPE cells face substantial oxidative stress due to their unique physiological roles and microenvironment. One primary source is their exposure to high ambient oxygen levels, driven by the retina’s exceptional metabolic demands. The macula, in particular, receives some of the highest blood flows in the body, subjecting the RPE to oxygen partial pressures of 70–90 mm Hg [19]. This oxygen-rich environment inherently elevates the risk of oxidative damage. Additionally, the RPE performs the critical task of phagocytosing approximately 30,000 photoreceptor outer segments daily [20]. During this process, phagosomal NADPH oxidase and the peroxisomal β-oxidation of outer segment lipids generate intracellular H_2_O_2_ at levels comparable to those observed in activated macrophages [21], further amplifying oxidative stress [22]. Photo-oxidative stress from processing light for vision is perhaps the most unique and additional source of oxidative stress. Photo-oxidative stress represents another distinct challenge. The role of the RPE in processing light for vision exposes it to continuous photochemical damage, which directly contributes to oxidative injury in the retina, RPE, and choroid [23]. In response to these external stressors, the RPE itself produces high levels of physiological ROS because of its intense metabolic activity. Its dense mitochondrial network, which is essential for meeting energy demands, serves as a major endogenous source of ROS. While mitochondrial ROS participate in normal signaling, aging exacerbates their harmful effects: older mitochondria not only generate more ROS, but also render cells increasingly vulnerable to oxidative damage.

Drusen formation, a hallmark of early AMD, arises from the progressive accumulation of basal linear deposits (BLinD) and basal laminar deposits (BLamD) beneath the RPE. Proteomic analyses identify drusen as extracellular deposits rich in lipids, complement proteins (e.g., C3, C5b-9), and inflammatory mediators such as amyloid-β (Aβ) [24], with lipids constituting more than 40% of the drusen volume [25].

#### 2.3.1. Mitochondrial Dysfunction as a Central Driver of Oxidative Stress

Mitochondria are essential organelles responsible for adenosine triphosphate (ATP) production via oxidative phosphorylation. This process involves electron transfer through multi-subunit complexes (I to IV) of the electron transport chain (ETC), coupled with molecular oxygen reduction. Under physiological conditions, 2–5% of oxygen undergoes incomplete reduction, generating elevated ROS and superoxide [26]. Mitochondrial dysfunction critically underpins oxidative stress in the RPE, serving as a major source of ROS. This dysfunction plays a pivotal role in the pathogenesis of AMD through interconnected mechanisms: oxidative stress, mitochondrial DNA (mtDNA) damage, impaired autophagy/mitophagy, and chronic inflammation [27].

In AMD, ROS production is exacerbated [28], inducing oxidative damage to mtDNA—which lacks protective histones and resides near ROS-generating ETC complexes (Figure 1, part 3) [29]. Such damage is more extensive in AMD patients than in age-matched controls [28], impairing ETC subunit genes, reducing ATP synthesis, and amplifying ROS generation in a self-perpetuating vicious cycle [29]. Furthermore, mtDNA repair mechanisms (e.g., base excision repair, BER) exhibit reduced efficiency in AMD, promoting lesion accumulation [30].

Autophagy, particularly the selective clearance of damaged mitochondria via PINK1-Parkin-mediated mitophagy [31], is also compromised [32]. RPE-specific autophagy knockouts recapitulate AMD phenotypes, confirming this impairment. Mitophagy failure—linked to the dysregulation of regulators like NFE2L2 and PGC-1α—results in the accumulation of dysfunctional mitochondria, lipofuscin, and drusen, all pathological hallmarks of AMD [33]. Concurrently, damaged mitochondria activate the NLRP3 inflammasome [34], triggering the release of pro-inflammatory cytokines (e.g., IL-1β, IL-18) that exacerbate RPE degeneration [35].

#### 2.3.2. Lipofuscin Accumulation

Lipofuscin accumulates in the RPE as a byproduct of light-driven vitamin A recycling in the visual cycle. This fluorescent pigment, which is primarily composed of the bisretinoid *N*-retinyl-*N*-retinylidene ethanolamine (A2E) [36], progressively builds up with age and is implicated in AMD pathogenesis (Figure 1, part 4) [37], although its precise role remains under investigation [38]. The essential role of the RPE in the phagocytosis of lipid-rich photoreceptor outer segments becomes compromised over time, leading to the detection of cytotoxic lipofuscin deposits via fundus autofluorescence imaging. A2E, a condensation product of all-trans retinal (atRAL), exacerbates oxidative stress by sensitizing RPE cells to blue light, which generates singlet oxygen and superoxide radicals that overwhelm endogenous antioxidant defenses [39]. These reactive species damage mitochondrial DNA, disrupt electron transport chain function—as evidenced by a 60% reduction in cytochrome c oxidase activity in AMD-affected RPEs [40]—and ultimately trigger apoptosis [41]. Concurrently, the lipid peroxidation of PUFAs generates toxic aldehydes such as 4-hydroxynonenal (4-HNE) and malondialdehyde (MDA). These byproducts form adducts with proteins and DNA, disrupting cellular homeostasis [42]. Notably, MDA accumulation in AMD modifies endogenous molecules, creating oxidation-specific epitopes that contribute to disease progression. The Y402 polymorphism in complement factor H (CFH), a major genetic risk factor for AMD, markedly impairs the ability of CFH to bind MDA, providing a mechanistic link between genetic susceptibility and oxidative damage [43]. In advanced AMD, these peroxidation products accumulate within drusen and the Bruch’s membrane (BrM), exacerbating local inflammation and impairing nutrient transport to photoreceptors, thereby driving degenerative changes.

## 3. Inflammation and Immune Dysregulation

Inflammation and immune dysregulation play central roles in AMD, particularly in its early pathogenesis. Drusen, the hallmark extracellular deposits of dry AMD, contain proinflammatory components such as apolipoprotein E, coagulation factors, acute-phase proteins, IgG, and complement activators, which collectively signify localized inflammation as a critical driver of disease initiation [44]. Convergent evidence underscores inflammatory cascades [45] and immune imbalance [46] as key mechanisms promoting drusen formation and AMD progression [47]. These processes involve the accumulation of extracellular deposits, regional immune activation, and the recruitment of microglia and macrophages to subretinal and choroidal regions, amplifying tissue damage.

**Figure 2:**
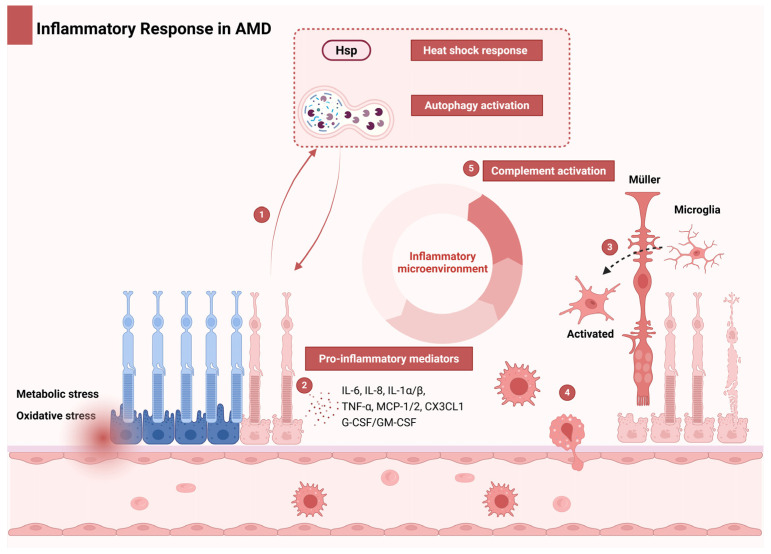
Inflammatory cascade and immune dysregulation in AMD. Under oxidative/metabolic stress, compromised retinal neurons and RPE cells initiate an inflammatory cascade. **Part 1**: Early adaptation: HSPs and autophagy provide transient protection. **Part 2**: Proinflammatory microenvironment: Chronic stress in aging retinas overwhelms protective mechanisms, inducing cellular senescence/apoptosis. Senescent cells establish a self-perpetuating microenvironment via senescence-associated proinflammatory mediators, including IL-6, IL-8, TNF-α, IL-1α/β, MCP-1/2, CX3CL1, and G-CSF/GM-CSF. **Part 3**: Local amplification: Proinflammatory mediators activate resident microglia, Müller cells, and RPE while recruiting choroidal macrophages. **Part 4**: Complement dysregulation: Local inflammation amplifies complement system activity. **Part 5**: Systemic engagement: Exceeding adaptive thresholds triggers systemic innate immune activation and complement amplification. This feedforward loop drives AMD progression through oxidative damage, chronic immune activation, and complement-mediated tissue injury. Abbreviations: AMD, age-related macular degeneration; G-CSF, granulocyte colony-stimulating factor; GM-CSF, granulocyte–macrophage colony-stimulating factor; HSP, heat shock protein; IL, interleukin; MCP-1/2, monocyte chemoattractant protein-1/2; RPE, retinal pigment epithelium; TNF-α, tumor necrosis factor-alpha.

The aging retina increasingly accumulates oxidative damage, which is marked by elevated protein oxidation and lipid peroxidation byproducts [48]. Under oxidative or metabolic stress, retinal neurons and RPE cells experience structural and functional compromise, triggering a graded immune response (Figure 2). Initially, adaptive mechanisms such as heat shock protein induction and autophagy activation attempt to mitigate damage and restore homeostasis (Figure 2, part 1). However, chronic stress—characteristic of aging—overwhelms these defenses, driving cellular senescence or apoptosis.

### 3.1. Immune Cell Involvement

Senescent cells secrete proinflammatory mediators, including IL-6, IL-8, TNF-α, IL-1α/β, MCP-1/2, CX3CL1, and granulocyte/macrophage colony-stimulating factor (G-CSF/GM-CSF), which establish a proinflammatory microenvironment (Figure 2, part 2) [49]. These cytokines activate resident retinal immune cells (microglia, Müller cells, and RPE) (Figure 2, part 3) and recruit choroidal macrophages (Figure 2, part 4). Immune cells, particularly retinal microglia and infiltrating macrophages, critically contribute to AMD pathology. Single-cell RNA sequencing studies have identified distinct microglial subpopulations in AMD retinas, revealing a shift toward proinflammatory M1-like states that exacerbate disease progression [50]. Resident microglia, which are activated by drusen components and oxidative stress, adopt this inflammatory phenotype, releasing cytokines such as IL-1β, IL-6, and TNF-α [51]. These mediators impair RPE function and promote CNV in neovascular AMD. Concurrently, peripheral macrophages are recruited to the retina via chemokine ligand 2 (CCL2)-CCR2 signaling, amplifying inflammatory cascades and perpetuating a cycle of tissue damage and immune activation. This interplay between resident and recruited immune cells creates a self-sustaining inflammatory milieu, driving retinal degeneration and advancing AMD pathology.

### 3.2. Complement System Dysregulation

When tissue stress surpasses local adaptive thresholds, systemic cytokine release mobilizes innate immunity, including complement amplification (Figure 2, part 5), to mediate tissue remodeling through parainflammation—a homeostatic response that becomes dysregulated in aging retinas [52]. This disruption transforms a protective mechanism into a pathological driver, exacerbating AMD progression by sustaining chronic inflammation, promoting further immune cell infiltration, and disrupting retinal–choroidal crosstalk.

**Figure 3:**
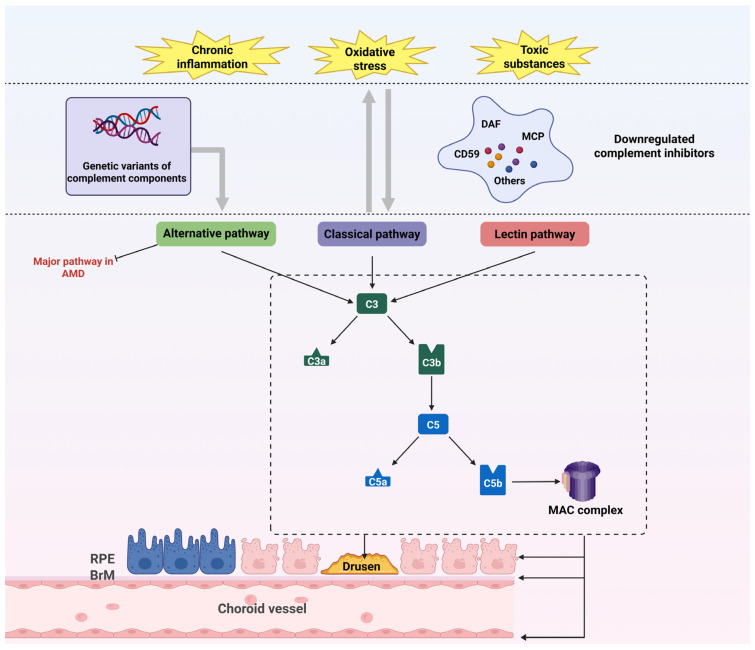
Complement system dysregulation in AMD. Oxidative stress, chronic inflammation, and visual cycle byproducts downregulate complement inhibitors (DAF, MCP, CD59), enabling uncontrolled complement activation. Genetic susceptibility amplifies complement dysregulation. Subsequent deposition of C3/C5 within drusen and C5b-9 MAC drives structural damage to RPE, BrM, and choriocapillaris. A self-perpetuating cycle emerges wherein complement activation exacerbates oxidative stress, inflammation, and toxin accumulation. Abbreviations: AMD, age-related macular degeneration; BrM, Bruch’s membrane; DAF, decay-accelerating factor; MAC, membrane attack complex; MCP, membrane cofactor protein; RPE, retinal pigment epithelium.

Complement system dysregulation is a cornerstone of AMD pathogenesis and is driven by chronic inflammation and innate immune hyperactivity. The alternative complement pathway dominates this process, although all three pathways (classical, lectin, alternative) contribute to host defense under normal conditions (Figure 3). In AMD, oxidative stress and visual cycle byproducts trigger RPE and choroidal cells to downregulate complement inhibitors such as decay-accelerating factor (DAF), membrane cofactor protein (MCP), and CD59, enabling pathological complement activation in host tissues [53]. Histopathological studies have confirmed this mechanism, revealing that the complement components C3 and C5 within drusen and the C5b-9 membrane attack complex (MAC) are deposited in the aged Bruch’s membrane (BrM) and choriocapillaris (CC) of AMD donors [46]. Genetic evidence further solidifies this link: genome-wide association studies (GWAS) implicate over 50 AMD-associated loci, with complement genes (*CFH*, *CFI*, *CFB*, *C3*, *FB/C2*) accounting for a significant proportion of disease heritability (Table 1) [54].

The *CFH* Y402H polymorphism (rs1061170), present in ~50% of AMD patients, exemplifies this dysregulation. This variant reduces CFH binding affinity for glycosaminoglycans and C-reactive protein (CRP) [55], impairing its ability to inhibit alternative pathway C3 convertase [56] and leading to uncontrolled C3b deposition and MAC formation at the RPE-BrM interface (Figure 3) [57]. These events coincide with lipoprotein accumulation in drusen, amplifying oxidative and inflammatory damage [58]. In addition to its regulatory role, CFH exerts cell-autonomous protective effects: endogenous CFH in RPE cells preserves transcriptional and metabolic homeostasis under oxidative stress [59], while exogenous CFH administration mitigates oxidized lipid-induced RPE injury [60]. The *CFH* H402 variant also disrupts MDA binding and lipid metabolism [43], as demonstrated in aged mice fed high-fat diets that develop AMD-like pathology with genotype-dependent lipoprotein dysregulation in ocular tissues and plasma [61].

Complement amplification is further exacerbated by gain-of-function variants in *CFB* (enhancing alternative pathway activity) and *C3* (rs2230199 R102G, increasing C3b production), which drive MAC-mediated RPE lysis, photoreceptor degeneration, and chronic inflammation (Figure 3) [62]. These findings underscore complement dysfunction as a multifactorial driver of AMD, extending beyond immune activation to disrupt metabolic homeostasis, exacerbate oxidative stress, and synergize with lipid dysregulation. Overall, complement dysregulation connects genetic susceptibility, inflammatory cascades, and tissue remodeling, positioning complement dysregulation as both a biomarker and therapeutic target in AMD.

## 4. Lipid Metabolism and Drusen Formation

**Figure 4:**
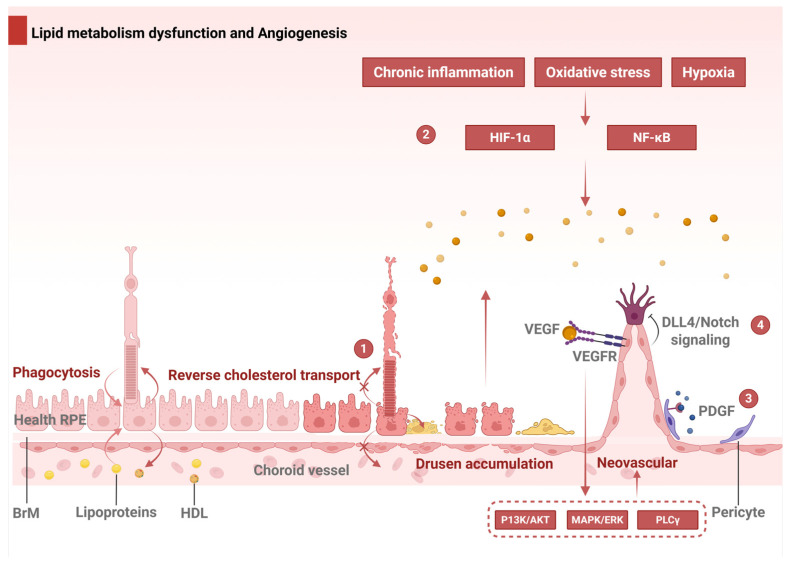
Dysregulated lipid metabolism and angiogenic signaling in AMD. **Part 1:** Cholesterol homeostasis and pathological RCT forming sub-RPE deposits. **Part 2:** Hypoxia/oxidative stress activates HIF-1α/NF-κB, inducing VEGF-driven CNV. **Part 3:** PDGF amplifies CNV through pericyte recruitment and vessel destabilization. **Part 4:** VEGF-induced Dll4/Notch signaling regulates endothelial tip–stalk patterning; dysregulation causes aberrant sprouting. Abbreviations: RPE, retinal pigment epithelium; BrM, Bruch’s membrane; RCT, reverse cholesterol transport; VEGF = A, vascular endothelial growth factor A; HDL, high-density lipoprotein; VEGFR, VEGF receptor; PDGF, platelet-derived growth factor; Dll4, Delta-like ligand 4.

The RPE secretes cholesterol-rich lipoproteins containing apolipoproteins B (ApoB) and E (ApoE) into BrM [63], which is derived from ingested circulating lipoproteins or phagocytosed photoreceptor outer segments (Figure 4, Part 1) [64]. Under normal physiological conditions, the RPE recycles cholesterol to photoreceptors or exports it via reverse cholesterol transport mediated by ApoA-I [65], forming high-density lipoprotein (HDL) particles [66]. However, impaired reverse cholesterol transport drives the RPE to secrete ApoB100-containing lipoproteins into BrM [67], initiating lipid accumulation akin to atherosclerotic processes [24]. Soft drusen and BLinD emerge when aged BM and the choroidal endothelium fail to clear these effluxed metabolites, leading to sub-RPE deposits (Figure 4, Part 1) [64].

Genetic studies underscore lipid metabolism’s centrality to AMD, with variants in *LIPC*, *CETP*, *ABCA1*, and *APOE* influencing disease risk [63]. The *APOE-ε4* allele reduces the drusen burden but exacerbates complement activation, whereas *APOE-ε2* promotes cholesterol retention and increases hydraulic resistance in BrM [68]. The oxidative modification of lipoproteins within drusen generates lipid peroxides [69] and hydroxyapatite deposits coated with inflammatory proteins [70], triggering angiogenesis [71] and inflammation [72]. Advanced lipid peroxidation end products (ALEs) further accumulate in drusen, BrM, and lipofuscin [38], destabilizing proteins and inducing apoptosis in photoreceptors and RPE cells40 [36].

Diet critically modulates lipid dynamics in AMD. Omega-3 PUFAs (ω-3 PUFAs), such as docosahexaenoic acid (DHA), which is abundant in photoreceptors, exert anti-inflammatory and antioxidant effects. The AREDS2 trial demonstrated that ω-3 PUFA supplementation with antioxidants reduces advanced AMD progression by 25% [73]. Conversely, diets high in trans and saturated fats increase AMD risk, with BrM lipid profiles reflecting dietary intake rather than photoreceptor-derived sources [64]. Therapeutic strategies targeting lipid deposition, such as intravitreal L-4F (an ApoA-I mimetic), restore the BrM ultrastructure in preclinical models by clearing lipids and complement deposits [74]. However, clinical trials on statins have shown inconclusive benefits, and a Cochrane review revealed insufficient evidence to support their use in AMD prevention [75]. Overall, dysregulated lipid metabolism connects genetic predispositions, oxidative stress, and inflammatory pathways, positioning it as a pivotal therapeutic target in AMD.

## 5. Angiogenesis

### 5.1. VEGF Signaling: A Central Driver

VEGF-A drives pathological angiogenesis in wet AMD primarily by binding to VEGF receptor 2 (VEGFR2) on endothelial cells, activating downstream pathways such as the PI3K/Akt, MAPK/ERK, and PLCγ pathways (Figure 4, part 2). These pathways stimulate endothelial proliferation, migration, and lumen formation [76], culminating in CNV [77]. Hypoxia and inflammation further amplify VEGF-A expression through the transcriptional regulators HIF-1α and nuclear factor kappa-light-chain-enhancer of activated B cells (NF-κB), directly linking microenvironmental stress to aberrant vessel growth (Figure 4, part 2) [77]. Additional VEGF family members, including VEGF-C and VEGF-D, exacerbate CNV progression by interacting with VEGFR-2 and VEGFR-3, enhancing vascular permeability and neovessel formation. Elevated VEGF-C/D levels in the RPE and vitreous of AMD patients underscore their pathogenic contributions. Co-regulators such as platelet-derived growth factor (PDGF) (Figure 4, part 3) and angiopoietin-2 (Ang-2) further modulate CNV by recruiting pericytes and destabilizing the mature vasculature, respectively. Concurrently, dysregulated Notch signaling disrupts endothelial cell patterning. VEGF-A induces Delta-like ligand 4 (Dll4) in tip cells, which activates Notch receptors in adjacent stalk cells, suppressing VEGFR2 expression to establish a balanced tip–stalk cell distribution (salt-and-pepper pattern) (Figure 4, part 4). In AMD, aberrant Notch signaling disrupts this balance, resulting in excessive sprouting and disorganized vascular networks.

### 5.2. Inflammation and the Immune–Vascular Axis

Chronic inflammation is a hallmark of wet AMD pathogenesis. RPE cells secrete VEGF-A under oxidative stress or complement activation (e.g., by C3a and C5a), directly fueling CNV development [78]. Complement fragments C3a and C5a, detected in AMD-associated drusen, are correlated with increased VEGF expression and CNV severity. NLRP3 inflammasome activation in the RPE and immune cells (e.g., microglia and macrophages) drives the release of IL-1β and IL-18, perpetuating a pro-angiogenic microenvironment. Genetic predispositions, such as the *CFH* Y402H variant, exacerbate systemic inflammation [79] (e.g., elevated IL-6, TNF-α) and AMD risk [77]. Immune cells play dual roles: proinflammatory macrophages secrete VEGF-A to promote CNV, while alternatively activated subsets may mitigate angiogenesis. Single-cell analyses revealed upregulated IL-4/IL-13 signaling in AMD endothelial cells, suggesting that VEGF-independent angiogenic pathways may expand therapeutic targets.

### 5.3. Metabolic Reprogramming in Endothelial Cells

Metabolic dysregulation in endothelial cells is emerging as a critical contributor to CNV [80]. AMD endothelial cells exhibit altered glycolysis, oxidative phosphorylation, and amino acid metabolism. Glycolysis dominates ATP production, with the YAP-mediated upregulation of PFKFB3 enhancing glycolytic flux and CNV progression. The pharmacological inhibition of glycolysis (e.g., 3PO) reduces CNV in preclinical models. Glutamine and arginine metabolism support TCA cycle activity, redox balance, and nitric oxide synthesis, whereas fatty acid oxidation (FAO) fuels tip cell sprouting—processes inhibited by etomoxir (a CPT1 inhibitor) or pitavastatin. Mitochondrial ROS and calcium signaling further modulate angiogenic responses. Metabolomic profiling identifies distinct lipidomic signatures (e.g., glycerophospholipids, sphingolipids) and altered glucose metabolism (e.g., reduced α-ketoglutarate) in AMD patients, highlighting systemic metabolic dysregulation as both a biomarker and therapeutic avenue.

## 6. The Collapse of Neurovascular–Immune Microenvironment Crosstalk

### 6.1. Molecular Mechanisms of Blood–Retinal Outer Barrier Disruption

The outer blood–retina barrier (oBRB) comprises tight junctions (TJs) within the RPE, which act to regulate and filter the molecular movement of solutes and nutrients from the choroid to the subretinal space. The integrity of the oBRB relies on the coordinated function of three interconnected components: the RPE, CC, and BrM [81]. Disruption of the oBRB is particularly relevant to the pathology of AMD [82], which occurs due to photoreceptor/RPE/BrM/CC complex alterations [83].

Within the oBRB, TJs play a critical role in maintaining the barrier between the subretinal space and the CC. However, studies have shown that aging can compromise this barrier, as evidenced by a localized reduction in the TJ protein zona occludens-1 (ZO-1) in the RPE cells of aged mice [84], indicating TJ disruption in the oBRB. This disruption may be driven by oxidative stress, which has been shown to alter the distribution of ZO-1 and occluding [85], as well as cadherin in adherent junctions [86]. Notably, Cho et al. demonstrated that the oxidative stress-induced downregulation of thioredoxin-interacting protein (TXNIP) may contribute to TJ dysfunction by activating Src kinase, leading to the destabilization of ZO-1 in RPE cells [87]. These findings collectively suggest that oxidative stress is a potential driver of TJ disruption in the oBRB rather than merely exacerbating an existing condition, highlighting its central role in the breakdown of retinal barrier function.

In addition to oxidative stress, inflammation has been implicated as a significant contributor to the disruption of TJs in the oBRB. Previous studies have shown that inflammatory mediators, such as TNF-α [88], can directly influence the distribution and expression of key junctional proteins in RPE cells through the activation of p38 MAPK signaling pathways [89]. Moreover, van der Wijk et al. demonstrated that TNF-α could increase the permeability of the BRB in vitro, a process mediated by cAMP but independent of Rho/Rac signaling [90]. Moreover, in the context of an aged or damaged RPE, monomeric CRP can reach the apical side of the RPE, where it amplifies the proinflammatory microenvironment and further exacerbates barrier dysfunction [91]. Together, these findings highlight the multifaceted role of inflammation in driving oBRB disruption, particularly under conditions of RPE compromise.

The accumulation of abnormal substances within RPE cells could also lead to TJ disruption. Oxidized LDL, which is abnormally deposited due to lipid metabolism disorders, can mediate oxidative stress and inflammation in the RPE through the canonical Wnt pathway, leading to the disruption of ZO-1 arrangement at intercellular junctions [92]. Like Alzheimer’s disease, the deposition of Aβ is also a common pathologic feature of AMD [93]. ZO-1 and occludin are markedly attenuated [94] and lose their integrity as TJs in the RPE when intracellular and extracellular Aβ accumulate [95].

Disruption of the RPE-RPE interaction is often a precursor to epithelial–mesenchymal transition (EMT). Persistent activation of the transforming growth factor β (TGF-β) pathway triggers the loss of adhesive properties and polarity in RPE cells, inducing a mesenchymal phenotype through EMT [96]. In healthy eyes, the RPE secretes low levels of VEGF to suppress endothelial cell apoptosis and sustain the integrity of the CC-associated endothelium. Initiated by RPE dysfunction [83], dry AMD is characterized by significant thinning of the CC [97], with degenerative capillary endothelial cells, altered pericyte distribution [98], and reduced endothelial fenestration [99]. In addition to the RPE, intimate pericyte–endothelial connections remain fundamentally important for vascular integrity. Mediated by the angiopoietin-1/Tie2 signaling pathway, these connections could stimulate angiogenesis and shield retinal neurons from ischemic injury [100]. However, these interactions become weak and disrupted during aging, leading to capillary dropout, vascular abnormalities, and oBRB compromise [101]. In contrast, wet AMD initially involves choroidal vascular loss that disrupts the photoreceptor–RPE–Bruch’s membrane–choriocapillaris complex [83]. A hemodynamic model of AMD revealed that the CC underwent reduced perfusion and vascular density, leading to impaired nutrient exchange, oxidative stress, and inflammation [102]. Furthermore, in an inflammation-prone setting with a buildup of complement factors [46], along with pro-inflammatory agents such as CRP [103], the CC is vulnerable to dysfunction, making the adjacent RPE hypoxic. Thus, the hypoxia-induced RPE due to diminished blood flow may enhance VEGF secretion, thereby triggering CNV [104].

BrM is a critical extracellular matrix layer situated between the RPE and the CC and plays a vital role in maintaining the integrity of the oBRB. In AMD, there are several structural and functional changes in the BrM, such as waste accumulation, thickening, and calcification, which disrupt the BRB by impairing permeability and elasticity [105]. Additionally, in wet AMD, the BrM constitutes an important obstacle to pathological CNV [106]. However, an increased expression of MMP-9, which attacks the architecture of the BrM [107], has been demonstrated in AMD [108]. The MMP-9-mediated degradation of BrM structural proteins enables endothelial cell migration in angiogenesis, thereby initiating CNV formation [106].

### 6.2. Cascade Mechanisms of Photoreceptor Degeneration

Photoreceptor degeneration is a central event in the progression of AMD, leading to irreversible vision loss [109]. This degenerative process is not a singular event, but rather a cascade of interconnected molecular and cellular events initiated by oxidative stress and amplified through inflammatory responses, metabolic dysregulation, and cellular apoptosis.

**Figure 5:**
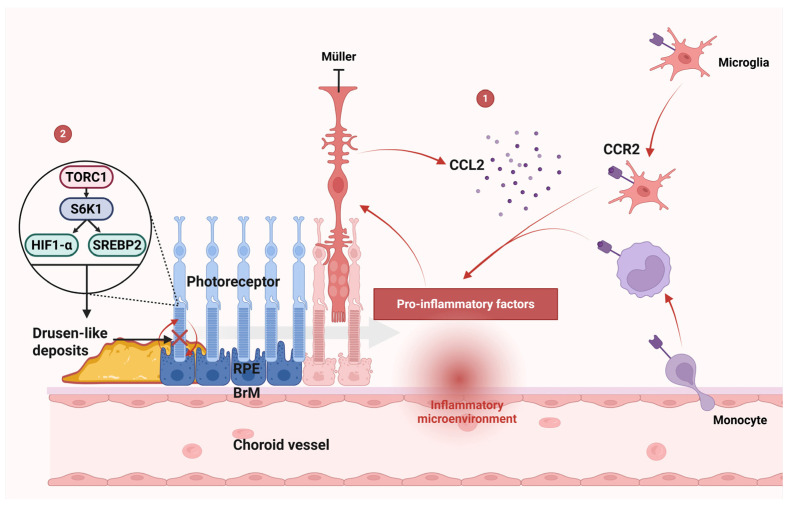
Mechanisms of photoreceptor degeneration. **Part 1: CCL2-CCR2 axis:** CCL2 recruits CCR2+ monocytes/microglia to sites of retinal injury. Infiltrating cells release proinflammatory cytokines, driving RPE and photoreceptor degeneration. Müller cell activation amplifies this cascade through CCL2 production, establishing a feedforward inflammatory loop. **Part 2: mTORC1 pathway:** mTORC1 hyperactivity contributes to drusen-like deposit formation, disrupting RPE-photoreceptor metabolic exchange and accelerating AMD progression. Abbreviations: AMD, age-related macular degeneration; CCL2, C-C motif chemokine ligand 2; CCR2, C-C chemokine receptor type 2; HIF1-α, hypoxia-inducible factor 1-alpha; mTORC1, mechanistic target of rapamycin complex 1; RPE, retinal pigment epithelium; SREBP2, sterol regulatory element-binding protein 2.

As established in Section 2, oxidative stress in the RPE, driven by mitochondrial dysfunction and lipid peroxidation, serves as the primary initiator of photoreceptor degeneration in AMD. This oxidative damage not only impairs RPE function but also sets the stage for subsequent inflammatory and degenerative processes. The CCL2-CCR2 axis plays a significant role in the pathogenesis of AMD by driving chronic inflammation and contributing to photoreceptor degeneration. CCL2, also known as monocyte chemoattractant protein-1 (MCP-1), is a key chemokine that recruits CCR2-positive monocytes [110] and microglia [111] to sites of retinal injury or inflammation (Figure 5, part 1) [112]. In AMD, CCL2 is often upregulated in response to oxidative stress [113] and other pathological stimuli [114], leading to the infiltration of these immune cells into the subretinal space.

Once recruited, CCR2-positive monocytes and microglia release proinflammatory cytokines, exacerbating tissue damage and promoting the degeneration of RPE cells and photoreceptors (Figure 5, part 1) [112]. This inflammatory cascade is further amplified by the activation of Müller cells, which produce CCL2 in response to macrophage infiltration [115]. The expression of CCL2 by Müller cells could further promote the infiltration of monocytes/microglia [116]. In addition, activated Müller cells may also reduce the secretion of neurotrophic factors [115], further compromising photoreceptors’ survival.

Targeting this axis, either by inhibiting CCL2 or blocking CCR2, has emerged as a promising therapeutic strategy to mitigate inflammation, protect retinal cells, and potentially slow the progression of AMD. Previous studies have shown that genetic knockout of the CCR2 gene can impair the recruitment of microglia [110] and reduce photoreceptor apoptosis [111], highlighting the detrimental role of the CCL2-CCR2 axis in AMD progression. Florian Sennlaub et al. reported that retinal tissues constitutively express CX3CR1, which suppresses CCL2 production and inhibits CCR2+ monocyte infiltration, serving as a critical regulator of inflammatory responses and neurodegenerative processes in AMD [112]. By disrupting this chemokine-receptor interaction, it may be possible to reduce the chronic inflammatory environment that drives AMD pathology.

The CCL2-CCR2 axis is just one of several interconnected pathways between oxidative stress and inflammatory pathways. Oxidative stress can directly trigger activation of the NF-κB pathway, resulting in an elevated production of multiple proinflammatory factors and the generation of a pro-inflammatory environment [117]. Moreover, the activation of Cyclic GMP-AMP Synthase–Stimulator of Interferon Genes (cGAS-STING) signaling, which is a key cytosolic DNA sensor system in innate immunity, was also detected in the context of oxidative stress-induced retinal degeneration [118]. DNA from oxidative stress-exposed cells strongly activate STING signaling [119], subsequently inducing type I interferon production through IRF3/IRF7 while simultaneously promoting inflammatory cytokine expression via NF-κB pathway activation [120].

Oxidative stress and inflammation contribute significantly to the progressive dysfunction of RPE cells. Oxidative stress induces damage to mitochondrial DNA integrity, lipid membranes, and cellular proteins within RPE cells [121], impairing their normal function. This mitochondrial damage can lead to a loss of mitochondria, which in turn alters the metabolic and differentiation state of the RPE [122]. Importantly, RPE cells play a critical role in supporting photoreceptor health by facilitating glucose transport via GLUT-1 glucose transporters [123]. However, oxidative stress and inflammation disrupt this supportive function. mTORC1 activation is subsequently required for the survival of metabolically stressed photoreceptors [124]. Activated mTORC1 can stimulate glycolysis, the pentose phosphate pathway, and lipid biosynthesis through the activation of HIF1-α and SREBP2 mediated by S6K1 (Figure 5, part 2) [125]. In retinitis pigmentosa, the activation of mTORC1 in cones promotes long-term cone survival in mice, with an increased utilization of glucose and increased levels of the key metabolite NADPH [124]. However, activated mTORC1 in photoreceptors has been linked to the formation of drusen-like deposits in AMD (Figure 5, part 2) [126]. These deposits interfere with the normal exchange of nutrients and waste products between the RPE and photoreceptors, further exacerbating RPE dysfunction and contributing to the progression of AMD.

The synergistic interplay of oxidative stress, inflammation, and metabolic dysfunction drives the progressive degeneration of photoreceptors through diverse cell death mechanisms, including apoptosis [127], necroptosis [128], ferroptosis [129], and dysregulated autophagy [130]. This culminates in the final step in the degenerative process, leading to permanent structural and functional deficits in the retina.

These advances in elucidating the cellular and molecular pathophysiology of AMD have catalyzed the development of multiple targeted therapeutic strategies. These include both clinically validated interventions and emerging treatment modalities that show considerable promise in preclinical and early-phase clinical studies.

## 7. Intervention Strategies in the Era of Precision Medicine

Despite these advances, current therapies targeting key pathogenic pathways, notably angiogenesis and complement dysregulation, remain limited by interpatient variability in treatment response and non-negligible adverse effects. To overcome these challenges, innovative therapeutic strategies are rapidly emerging, including epigenetic modulators, gut–retina axis interventions, and regenerative medicine approaches, which hold transformative potential for AMD management.

### 7.1. Limitations and Breakthroughs of Current Therapies

#### 7.1.1. Wet AMD

In recent years, anti-VEGF therapies have transformed wet AMD treatment, effectively preventing and reducing CNV in patients [131]. Nevertheless, a proportion of wet AMD cases demonstrate resistance to anti-VEGF agents, necessitating repeated intravitreal injections.

A previous study revealed that anti-VEGF therapy-resistant vasculature was supported by pericytes and was immunohistochemically positive for NG2, PDGFRβ, and αSMA in malignant melanoma [132]. In wet AMD, CD34+ progenitor cells exhibit plasticity in vitro, differentiating into either VWF+ endothelial cells or NG2+ pericytes [133]. However, a correlation between the refractory treatment response to anti-VEGF injections and the expansion potential and differentiation characteristics of CD34+ cells was not detected because of the variability in the results collected from wet AMD patients [133].

Bioinformatics and network pharmacology analysis revealed that wet AMD patients with anti-VEGF resistance showed enhanced immune system activation alongside reduced antioxidant capacity [134]. SOD1 was pinpointed as a hub gene in treatment-resistant pathogenesis, which was negatively correlated with immune infiltration [134]. In addition, Zhuang et al. demonstrated through integrated proteomic–metabolomic analysis and volumetric lesion assessment that decreased YIPF3 levels may predict anti-VEGF therapy failure, showing significant associations with limited lesion regression material in wet AMD [135].

Moreover, the compensatory upregulation of alternative pro-angiogenic mediators [136], such as PDGF, fibroblast growth factor, placental growth factor, interleukins, and TGF-β [137], may occur following VEGF blockade [138], which is implicated in the development of anti-VEGF resistance. Faced with the challenge of anti-VEGF resistance, various treatment options have been explored, including steroid injections, laser therapy, shortened anti-VEGF treatment intervals, switching anti-VEGF agents, and topical medications, with varying outcomes [139].

#### 7.1.2. Dry AMD

For dry AMD, contemporary clinical interventions are directed mainly at complement cascade modulation to attenuate the inflammatory-mediated degeneration in advancing GA. Pegcetacoplan, the first treatment approved by the US Food and Drug Administration (FDA) for GA, is a synthetic molecule that selectively inhibits C3, leading to a comprehensive downregulation of the entire complement system [140]. In two multicenter, randomized, double-masked, sham-controlled, phase 3 trials (NCT03525613, OAKS; NCT03525600, DERBY), pegcetacoplan slowed GA lesion growth in both the monthly and bimonthly arms [141]. However, the pegcetacoplan treatment group showed elevated frequencies of ocular complications such as intraocular inflammation, incident neovascular AMD, and anterior ischemic optic neuropathy relative to control arms [141]. In addition, the American Society of Retina Specialists (ASRS) investigated cases of retinal vasculitis and retinal artery occlusion following the treatment of pegcetacoplan, with seven recorded cases occurring within the first half-year post-approval [142]. For some patients, the associated adverse event risks could potentially offset the mild benefits of modestly slowing lesion progression [143].

The treatment effect of pegcetacoplan might depend on the specific GA phenotype. Using a deep learning model to illustrate the treatment effect of pegcetacoplan, Irmela Mantel et al. reported that extrafoveal lesions demonstrated greater treatment effects and therapeutic response in the foveal region compared to peripheral retinal areas [144], which corresponds well with a post hoc analysis of the OAKS and DERBY studies [145]. Moreover, deep learning-based OCT analysis has indicated that the difference in loss between the ellipsoid zone (EZ) and RPE strongly affects disease progression and the therapeutic response to pegcetacoplan, which might become an important criterion for the management of GA secondary to AMD [146].

In addition to pegcetacoplan, avacincaptad pegol, a C5 inhibitor, has also gained approval for treating GA secondary to AMD in adult patients [147]. In a randomized pivotal phase 2/3 trial (NCT02686658, GATHER1), twelve-month data revealed that avacincaptad pegol effectively reduced GA lesion growth rates in AMD [148]. And C5 inhibition may provide superior safety to pegcetacoplan by preserving C3 activity. Clinical data from the GATHER2 trial (NCT04435366) demonstrated significantly lower inflammatory risk with avacincaptad pegol—only one mild intraocular inflammation case reported over 24 months in this randomized, double-masked, sham-controlled phase 3 study [149].

### 7.2. Translational Potential of Emerging Therapeutic Targets

The interplay between epigenetic and metabolic pathways reveals critical molecular crosstalk, offering novel insights into AMD pathogenesis and treatment targets [150]. Retinal DNA methylation profiling has uncovered 87 significant gene–epigenome interactions in human samples, with specific DNA methylation alterations disrupting glutathione homeostasis and glycolytic flux pathways central to oxidative stress mitigation in early AMD [151]. Genome-wide methylation analyses further revealed an AMD-associated epigenetic dysregulation of SKI and GTF2H4, genes that govern TGF-β-mediated inflammation and transcription-coupled DNA repair mechanisms [152]. Additionally, analyses of metabolite quantitative trait loci (meQTLs) in AMD patients and controls have revealed significant associations involving metabolites and genes such as ASPM and LIPC, underscoring the potential role of glycerophospholipid metabolism in AMD pathogenesis [153]. Collectively, these findings position epigenetic-metabolic crosstalk as a dynamic driver of AMD pathology across disease stages.

**Figure 6:**
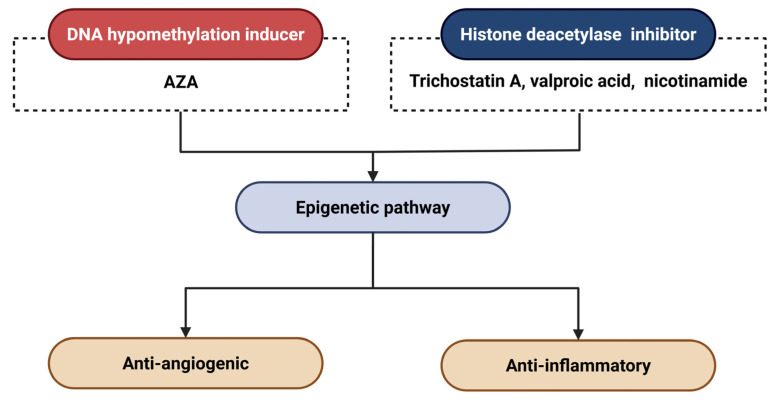
Epigenetic therapeutic strategies for AMD. Abbreviations: AZA, 5-aza-2′-deoxycytidine.

Epigenomic studies could inform the development of novel therapeutic strategies. Tiina Suuronen et al. reported that the induction of DNA hypomethylation with 5-aza-2′-deoxycytidine (AZA) could cause the overexpression of clusterin (CLU, 8p21.1) through the hypomethylation at its promoter region, influencing the pathogenesis of AMD through anti-angiogenic and anti-inflammatory effects (Figure 6) [154]. This can lead to the upregulation of clusterin (CLU, located at 8p21.1) by reducing methylation in its promoter region.

Histone deacetylase (HDAC) inhibitors represent promising therapeutic candidates for AMD, analogous to DNA hypomethylation-inducing agents, by modulating epigenetic pathways implicated in disease progression. Notably, trichostatin A, a pan-HDAC inhibitor, has been reported to suppress CNV through multiple mechanisms: it attenuates pro-angiogenic signaling, impedes CNV-associated wound healing responses, and inhibits pathological EMT in RPE cells [155]. In addition, pharmacological studies demonstrate that valproic acid [154] and nicotinamide [156] exert anti-angiogenic effects in AMD through HDAC inhibition, whereas sulforaphane confers photoprotection to RPE cells via HDAC suppression [157].

Furthermore, given the promising potential of RNA therapy for treating inherited retinal degeneration, RNA editing may pave the way for groundbreaking clinical advancements in epigenetic studies [158]. In addition to RNA therapy, induced pluripotent stem cells (iPSCs) are also expected to provide tools for performing functional assays to test key findings in epigenetic studies and serve as models for the development of therapeutics [159].

Aside from the advances in epigenetics, recent studies have highlighted the growing importance of systemic interactions in retinal diseases, particularly through the gut-retina axis. Accumulating data indicates possible participation of the gut-retina axis in AMD pathogenesis, driving the exploration of new therapeutic avenues.

A three-arm randomized controlled trial (NCT06391411) reported that micronutrient supplementation with lutein, zeaxanthin, and saffron correlated with enhanced visual acuity alongside a notable decrease in medium-chain fatty acid (MCFA) levels [160]. MCFAs act as important regulators of energy metabolism, membrane trafficking, and gene expression in various anaerobic bacteria. These results suggest that micronutrient supplementation may help restore the gut-retina axis, enhancing ocular outcomes in wet AMD patients [160].

Further research by Zhang et al. revealed metformin’s therapeutic potential in wet AMD through gut microbiota modulation and gut–retina axis regulation [161]. Specifically, metformin administration altered microbial composition, notably enhancing Bifidobacterium and Akkermansia populations, while elevating concentrations of butyrate, SCFAs, and cholic acid in fecal samples [161]. These microbial metabolites have been implicated in mediating the protective effects of metformin. For example, previous studies have shown that butyrate can inhibit neovascularization partially through the TXNIP/VEGFR2 signaling pathway [162]. Similarly, metformin-elevated SCFAs have been shown to stimulate GPR41 and GPR43 receptors on gut epithelial cells, activating mitogen-activated protein kinase (MAPK) signaling and inducing rapid chemokine/cytokine secretion, potentially influencing retinal inflammatory responses and angiogenic regulation [163]. For cholic acid, ursodeoxycholic acid (UDCA) was found to downregulate the expression of VEGF and have inhibitory effects on CNV in a preclinical study [164]. Together, these findings highlight the multifaceted role of gut microbiome-derived metabolites in mediating anti-wet AMD effects through the gut-retina axis.

In addition to the aforementioned therapies, emerging strategies such as dietary modifications, probiotics, fecal microbiota transplantation, and microbiome-targeted interventions may help normalize microbial composition, decrease inflammatory markers, and positively influence AMD outcomes [165].

### 7.3. Regenerative Medicine and Personalized Models

Stem cells possess the capacity to be directed toward ocular lineages, offering promise for vision rehabilitation and protection. Both embryonic-derived cells (ESCs) and induced pluripotent stem cells (iPSCs), along with tissue-resident adult stem cells, represent optimal cellular substrates for ophthalmologic regenerative applications [166].

Among these candidate cell sources, ESCs currently represent the most clinically advanced platform for ocular regeneration, particularly in RPE replacement therapies. Previous clinical studies involving ESC-derived RPE cell transplantation have shown promising safety profiles along with preliminary evidence of therapeutic potential [167,168,169,170,171], but the requirement of local immunosuppression is still one of the main downsides of this approach. Although the intraocular space has been described as immune-privileged, both surgical intervention and the pathological state of AMD can compromise the blood–retinal barrier’s integrity. Moreover, the RPE cells derived from ESCs exhibit significant histocompatibility complex disparities with host tissues. Despite these challenges, in a phase 1/2a, prospective, single-arm, interventional study (NCT02590692), clinical data demonstrated excellent safety and tolerability profiles of human ESC-RPE transplantation in advanced dry AMD patients through a median 3-year follow-up [172], providing promising evidence for the implant’s long-term viability.

While ESCs show clinical potential, alternative pluripotent stem cell sources face distinct challenges. Clinical trials using iPSC-RPEs, however, have yielded limited success in treating AMD. A single-patient study (UMIN000011929) reported the transplantation of an autologous iPSC-derived RPE patch on the native secreted basement membrane, without improvement in best-corrected visual acuity at the 12-month follow-up [173]. Beyond pluripotent stem cells, tissue-resident adult stem cells present unique advantages for ocular repair, though their clinical translation faces distinct hurdles [166]. These endogenous progenitors (e.g., retinal Müller glia-derived stem cells) have demonstrated multipotent differentiation capacity into functional photoreceptors and RPE cells in preclinical models [174]. Their intrinsic tissue compatibility theoretically eliminates immune rejection concerns associated with ESC/iPSC-derived transplants.

Several important challenges are hindering the clinical application of stem cell transplantation for AMD [166], including cell manufacturing, cell delivery, cell survival, physiological cell behavior, immunogenicity, and tumorigenicity [175]. Addressing these issues is imperative to ensure robust long-term functional integration and therapeutic efficacy.

Compared to single-cell grafts, the transplantation of complex 3D retinal tissues demonstrates superior functional integration potential. In primate models of retinal degeneration, hESC-derived retinal tissue differentiated into multiple retinal cell types and established synaptic connections with host tissue, offering clinically relevant insights for transplantation protocol optimization [176]. In a clinical trial (jRCTa050200027), allogeneic iPSC-derived retinal organoid sheets were safely transplanted in two advanced retinitis pigmentosa patients, demonstrating 2-year graft survival, increased retinal thickness, and stabilized visual function without serious adverse events, supporting further clinical investigation [177]. While clinical organoid research for AMD remains limited, this industry–academia collaboration demonstrates clinically translatable safety and efficacy, providing a technically and economically viable model.

The therapeutic advantages of 3D retinal tissues extend beyond structural integration to their paracrine signaling potential. Notably, 3D human retinal organoids secrete extracellular vesicles (EVs) with enhanced retinal specificity compared to conventional human umbilical cord MSC-derived EVs [178]. Proteomic analyses demonstrate that organoid-derived EVs are enriched in retinal function-related proteins and EV biogenesis proteins, suggesting their dual role in structural repair and microenvironment modulation [178]. This synergy between 3D tissue transplantation and EV-mediated signaling presents a comprehensive strategy for retinal regeneration, addressing both cellular replacement and pathological host environment challenges.

Apart from its therapeutic potential, 3D tissue culture modeling could also offer platforms for the high-throughput testing of therapeutic candidates and studies of gene interactions to improve models of complex genetic diseases [179]. Kevin Achberger et al. first presented the retina-on-a-chip (RoC), an innovative microphysiological platform mimicking human retinal architecture that provided vasculature-like perfusion and permitted the accurate simulation of physiological interactions between mature photoreceptor segments and the RPE in vitro [180]. The RoC has the potential to accelerate pharmaceutical innovation while offering novel perspectives on retinal disease pathogenesis. To create a high-throughput compatible drug screening platform that mimics physiological angiogenesis, Zhao et al. engineered a dual-reporter hPSC system for generating vascularized organoids in vitro [181]. This innovative model enables the quantitative assessment of VEGF inhibitors’ efficacy and the rapid screening of potential angiogenic modulators through bioluminescent detection [181]. The spectrum of therapeutic strategies reviewed here, from conventional to experimental, is comprehensively summarized (Table 2).

## 8. Future Perspectives: Integrating Multiomics and Artificial Intelligence

AMD is characterized by a multifactorial etiology involving genetic predisposition, metabolic dysregulation, and environmental influences. Despite significant advancements in understanding its pathogenesis, the heterogeneity of AMD and the variability in therapeutic responses remain substantial challenges. Recent progress in artificial intelligence (AI) and multiomics technologies has provided novel insights into the molecular mechanisms underlying AMD, offering transformative potential for both research and clinical practice.

AI-driven personalized approaches, including diagnosis, therapeutic decision-making, and prognostication, are reshaping the management of AMD. Deep learning algorithms have demonstrated exceptional proficiency in analyzing high-dimensional datasets derived from retinal imaging (e.g., optical coherence tomography (OCT)) and multiomics profiles, significantly enhancing diagnostic accuracy, sensitivity, and specificity for AMD [182]. Schranz et al. conducted a multicenter study of 230 wet AMD patients, which revealed significant quantitative associations between AI-quantified OCT fluid compartments and macular neovascularization characteristics, enabling accurate diagnosis and precision-guided therapeutic optimization [183]. Furthermore, long-term clinical outcomes may be improved by using AI algorithms to detect retinal fluid in AMD eyes, as a 10-year follow-on study showed that an AI algorithm achieved higher accuracy compared to retinal specialists, especially for intraretinal fluid [184]. In addition, the utilization of EV protein chips combined with machine learning methods identified ABCA1 as a biomarker for the early diagnosis of AMD, offering a promising new method for liquid biopsy diagnostics and improving the clinical diagnosis and management of AMD [185].

Beyond diagnostics, AI demonstrates superior precision compared to clinical specialists in personalizing anti-VEGF therapeutic selection. Several studies have focused on the AI-based prediction of anti-VEGF treatment outcomes to provide wet AMD patients with personalized medicine, including drug selection [186] and dose determination [187]. In a retrospective study, Moon et al. developed an attention-based generative adversarial network (GAN) model trained on 1684 OCT images to predict VEGF agent-specific anatomical treatment outcomes in patients with wet AMD, demonstrating the algorithm’s superior sensitivity over clinician assessment for short-term response prediction [186]. Notably, AI-driven biomarker discovery has identified novel therapeutic targets, including ferroptosis-associated diagnostic genes (e.g., *ABCA1*) and senescence-linked immune microenvironment signatures, which are correlated with distinct programmed cell death pathways [188] and disease progression trajectories [189]. Moreover, integrative AI models combining genetic data with imaging biomarkers have elucidated genotype–phenotype interactions, offering mechanistic insights into AMD pathogenesis while identifying candidate therapeutic targets [190].

For the prognostication of AMD progression, advanced techniques such as metadata-enhanced contrastive learning further refine feature extraction from longitudinal OCT datasets, improving risk stratification for GA conversion and wet AMD development [191]. Holland et al. demonstrated that a metadata-enhanced approach outperformed the alternative in AMD stage classification, subtype differentiation, and visual acuity prediction across two longitudinal datasets comprising 170,427 retinal OCT images from 7912 AMD patients [191]. These innovations collectively underscore AI’s transformative potential in transitioning AMD care from empirical protocols to data-driven, patient-specific strategies, although large-scale clinical validation remains imperative.

Building upon AI-driven advancements in AMD management, the synergistic integration of multiomics analytics and machine learning frameworks is emerging as a transformative paradigm for precision medicine. For example, Zhang et al. leveraged a deep subspace nonnegative matrix factorization (DS-NMF) algorithm to deconvolute multiomics profiles, reconstructing disease-associated gene expression trajectories that delineated AMD subtypes characterized by divergent immune pathway activation and macrophage infiltration patterns [192]. They further established a robust diagnostic model through multiple machine learning algorithms, including RF, AdaBoost, KNN, and SVM-RFE, bridging molecular heterogeneity to clinically actionable classification [192]. This multiomics-to-clinic pipeline exemplifies how AI can decode biological complexity to guide subtype-specific therapeutic development. In parallel, Jackson et al. integrated multiomics data with convolutional neural networks (CNNs) to uncover novel genetic and metabolic insights into retinal diseases and their systemic connections [193]. Such integrative approaches not only resolve mechanistic ambiguities, but also prioritize biomarkers with dual diagnostic and therapeutic relevance.

As these technologies continue to mature, they will undoubtedly reshape the landscape of AMD research and therapy, offering hope for improved patient outcomes and a deeper understanding of this complex disease. The convergence of multiomics and AI represents a paradigm shift in the study and management of AMD, heralding a new era of personalized and precision medicine in ophthalmology.

## 9. Criteria for Inclusion and Exclusion of the Literature

To ensure a comprehensive and unbiased review, a systematic literature search was conducted using the databases PubMed, Scopus, and Web of Science. These databases were selected for their extensive coverage of the medical and scientific literature relevant to AMD. The search utilized keywords including ‘age-related macular degeneration’, ‘AMD’, ‘oxidative stress’, ‘inflammation’, ‘lipid metabolism’, ‘immune dysregulation’, ‘angiogenesis’, and ‘molecular mechanisms’ to capture studies addressing the key pathophysiological processes discussed in this review. Studies were included if they were published in peer-reviewed journals, focused on the cellular and molecular mechanisms of AMD. Exclusion criteria encompassed studies not directly related to AMD, those not written in English, and purely clinical studies lacking mechanistic insights.

## Figures and Tables

**Table 1:** Major pathways involved in AMD, their roles, and associated genes.

Pathway	Role in AMD	Key Genes
Complement System	Immune dysregulation, drusen formation, inflammation	*CFH*, *CFI*, *C3*, *C5*, *C9*, *C2*, *CFB*, *CFD*
ECM Remodeling	Bruch’s membrane thickening, drusen formation	*TIMP-3*, *MMP-2*, *MMP-9*, *COL8A1*, *COL10A1*
Lipid Metabolism	Lipid accumulation in Bruch’s membrane, drusen formation	*APOE*, *LIPC*, *CETP*, *ABCA1*, *LPL*
Angiogenesis	Choroidal neovascularization in nAMD	*VEGF-A*, *FBLN5*
Oxidative Stress	RPE degeneration, photoreceptor death	*RAD51B*, *TNFRSF10A*, *ERCC6*
Autophagy/Phagocytosis	Impaired waste clearance, lipofuscin accumulation, inflammation	*ATG5*, *ATG7* (via animal models)

**Table 2:** Targeted therapeutic approaches for AMD.

Category	Therapy/Approach	Molecular Target(s)	Mechanism	Clinical Phase	Advantages	Limitations
**VEGF Inhibition**	Anti-VEGF [131] (Aflibercept/Ranibizumab)	VEGF-A, PIGF	Neutralizes angiogenic signaling	Phase 4 (post-marketing)	Gold standard for wet AMD	- Resistance development - Monthly injections required
**Complement Modulation**	Pegcetacoplan [140]	Complement C3	Pan-complement pathway suppression	Phase 3 (approved)	First FDA-approved GA therapy	- Wet AMD conversion - Retinal vasculitis risk [143]
	Avacincaptad pegol [149]	Complement C5	Terminal pathway inhibition	Phase 3 (approved)	Better safety profile	Slower effect vs. C3 inhibitors
**Epigenetic Therapies**	DNMT inhibitors (e.g., AZA [154])	DNA methyltransferases	Promoter hypomethylation (e.g., CLU gene)	Preclinical	Multimodal (anti-CNV, anti-inflammatory)	- High cytotoxicity - Non-specific effects - No ocular delivery system
	HDAC inhibitors (e.g., Trichostatin A [155])	HDAC classes I/II	Epigenetic modulation via histone deacetylase inhibition			
**Gut–Retina Axis**	Micronutrient supplementation (Lutein/Zeaxanthin [160])	MCFAs	↓ MCFAs → gut microbiome regulation	NCT06391411	Excellent safety	Moderate efficacy alone
	Metformin [161]	Gut microbiome alterations	↑ Microbial metabolites → anti-inflammatory, anti-angiogenic (e.g., ↑ Butyrate → TXNIP/VEGFR2 inhibition)	Preclinical	Oral administration	Variable microbiome response
**Regenerative Medicine**	ESC-RPE implants [166]	Embryonic-derived RPE replacement	Replaces damaged RPE with ESC/iPSC-derived RPE	Phase 1/2a	Long-term survival (3+ years)	- Requires immunosuppression - High cost
	iPSC-RPE patches [166]	Autologous RPE		Phase 1	No immune rejection	Poor visual outcomes in trials
	3D organoid platform [176]	-	3D tissue transplantation	Preclinical	Structural integration	Limitations of clinical practice

Abbreviations: AMD, aged-related macular degeneration; VEGF, vascular endothelial growth factor; PIGF, placental growth factor; CNV, choroidal neovascularization; FDA, US Food and Drug Administration; DNMT, DNA methyltransferase; AZA, 5-aza-2′-deoxycytidine; HDAC, histone deacetylase; MCFA, medium-chain fatty acid; ESC, embryonic-derived stem cells; iPSC, induced pluripotent stem cells; RPE, retinal pigment epithelium. ↑: upregulation; ↓: downregulation; →: leads to.

## Abbreviations

The following abbreviations are used in this manuscript:

AMD	Aged-related Macular Degeneration
VEGF	Vascular Endothelial Growth Factor
CNV	Choroidal Neovascularization
RPE	Retinal Pigment Epithelium
ROS	Reactive Oxygen Species
*HO-1*	Heme Oxygenase-1
*SOD2*	Superoxide Dismutase 2
Drp1	Dynamin-1-like protein
mtDNA	Mitochondrial DNA
GA	Geographic Atrophy
A2E	Bisretinoid N-retinyl-N-retinylidene ethanolamine
atRAL	All-trans retinal
4-HNE	4-hydroxynonenal
MDA	Malondialdehyde
CFH	Complement Factor H
BrM	Bruch’s Membrane
G-CSF/GM-CSF	Granulocyte/Macrophage Colony-Stimulating Factor
DAF	Decay-Accelerating Factor
MCP	Membrane Cofactor Protein
MAC	Membrane Attack Complex
CC	Choriocapillaris
CRP	C-Reactive Protein
CCL2	Chemokine Ligand 2
BLinD	Basal Linear Deposits
BLamD	Basal Laminar Deposits
ApoB	Apolipoproteins B
ApoE	Apolipoproteins E
HDL	High-Density Lipoprotein
ALE	Advanced Lipid Peroxidation End product
DHA	Docosahexaenoic Acid
NF-κB	Nuclear Factor Kappa-Light-Chain-Enhancer of Activated B Cells
PDGF	Platelet-Derived Growth Factor
Ang-2	Angiopoietin-2
FAO	Fatty Acid Oxidation
oBRB	Outer Blood–Retina Barrier
TJ	Tight Junction
ZO-1	Zona Occludens-1
TXNIP	Thioredoxin-Interacting Protein
EMT	Epithelial–Mesenchymal Transition
TGF-β	Transforming Growth Factor β
MCP-1	Monocyte Chemoattractant Protein-1
cGAS-STING	Cyclic GMP-AMP Synthase–Stimulator of Interferon Genes
PUFA	Polyunsaturated Fatty Acid
DNMT	DNA Methyltransferase
AZA	5-aza-2′-deoxycytidine
HDAC	Histone Deacetylase
MCFA	Medium-Chain Fatty Acid
CNN	Convolutional Neural Network
PIGF	Placental Growth Factor
FDA	US Food and Drug Administration
ASRS	American Society of Retina Specialists
EZ	Ellipsoid Zone
meQTLs	Metabolite Quantitative Trait Loci
iPSCs	Induced Pluripotent Stem Cells
SCFAs	Short-Chain Fatty Acids
MAPK	Mitogen-Activated Protein Kinase
UDCA	Ursodeoxycholic Acid
ESCs	Embryonic-Derived Cells
EVs	Extracellular Vesicles
hUC	Human Umbilical Cord
RoC	Retina-on-a-Chip
AI	Artificial Intelligence
OCT	Optical Coherence Tomography
DS-NMF	Deep Subspace Nonnegative Matrix Factorization
